# Malignant melanoma developing in a pre-existing cutaneous neurofibroma from a patient with neurofibromatosis type 1

**DOI:** 10.1016/j.jdcr.2024.09.022

**Published:** 2024-10-15

**Authors:** Stefanie L. Moye, Ariel Knowles, Travis Vandergriff, Lu Q. Le

**Affiliations:** a Department of Dermatology, University of Texas Southwestern Medical Center, Dallas, Texas; b Department of Dermatology, University of Virginia School of Medicine, Charlottesville, Virginia

**Keywords:** cutaneous neurofibroma, melanoma, neurofibromatosis type 1, NF1

## Introduction

Neurofibromatosis type 1 (NF1) is a genetic tumor predisposition syndrome affecting 1 in 3000 individuals. Loss of the tumor suppressor gene *NF1* and subsequent overactivation of RAS signaling lead to benign tumor development in the skin and nervous system. Characteristic skin findings include café-au-lait macules, axillary freckling, and cutaneous neurofibromas (cNFs). cNFs appear in nearly every patient with NF1 and do not undergo malignant transformation. While some individuals with NF1 develop other malignancies, the prevalence of melanoma and other skin cancers is not elevated within this population.^1^ Other studies have identified sporadic cases of cutaneous melanoma occurring in patients with NF1,^2^, ^3^, ^4^ and there is a case report of melanoma arising in the same location as the neurofibroma in a non-NF1 patient.^5^ We report a rare case of malignant melanoma developing in a pre-existing cNF in a patient with NF1.

## Case

**Fig 1:**
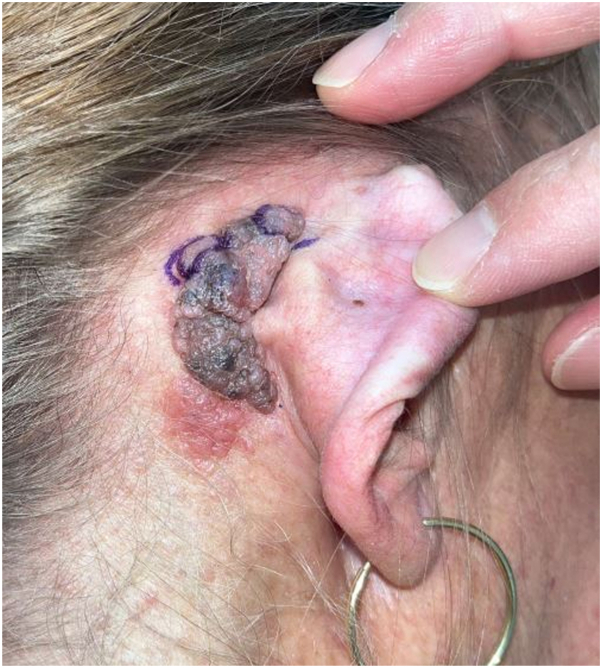
Clinical image. Malignant melanoma in the right retroarticular region.

A 56-year-old female with an established diagnosis of NF1 and a history of melanoma in situ and nonmelanoma skin cancer presented with a rapidly growing pigmentary lesion in her right postauricular region. The lesion was associated with pain and tenderness and had existed for ∼6 months at the time of presentation. The patient had a history of indoor and outdoor tanning and a family history of melanoma from her mother. Additionally, a few years prior, she had a wide local excision of melanoma on her right arm. Physical examination revealed a 2 × 3 cm cerebriform pink and dark-brown plaque in the right retroarticular region (Fig 1). The patient presented with multiple dermal neurofibromas on her back, a plexiform neurofibroma in the right pelvis, numerous café-au-lait macules, and axillary freckling. A shave biopsy of the plaque was taken.

**Fig 2:**
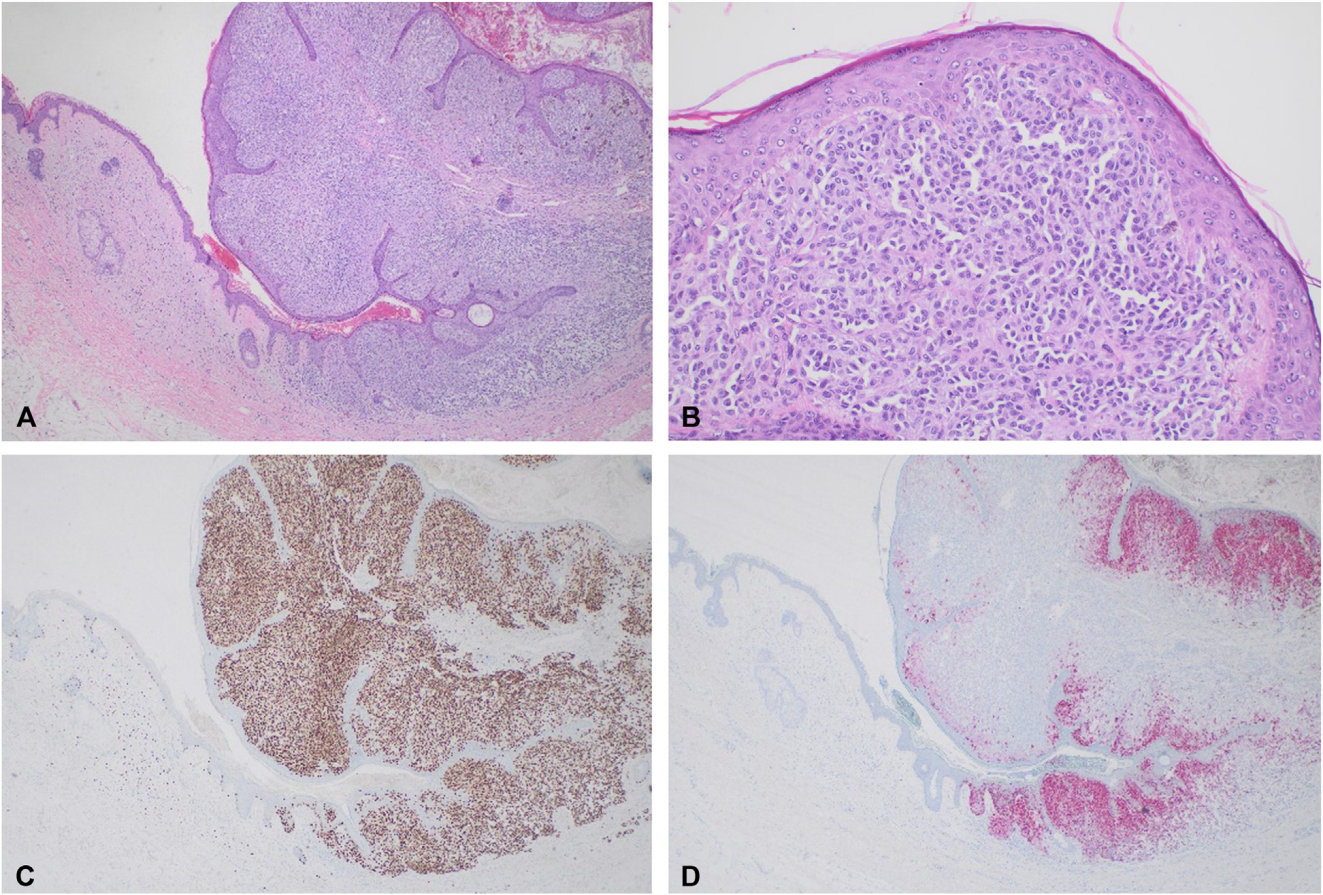
Histopathology of melanoma at the site of the pre-existing cNF. **A,** Hematoxylin and eosin stain which demonstrates an asymmetric and poorly circumscribed melanocytic neoplasm with junctional nests of varied size and shape. **B,** Higher power magnification reveals a proliferation of single melanocytes in the epidermis, with lentiginous hyperplasia and single melanocytes present in the spinous and granular cell layers in pagetoid extension. **C,** Immunohistochemistry staining for SOX10 which labels cells of neuroectodermal origin visualizes the spindle cells of the cNF (*left*) and melanocytes in the malignant melanoma (*right*). **D,** Immunohistochemistry staining for MART-1 demonstrates increased melanocyte proliferation in the epidermis. *cNF*, Cutaneous neurofibroma.

Microscopic evaluation revealed an asymmetric and poorly circumscribed melanocytic neoplasm formed by junctional nests that varied in size and shape (Fig 2, *A*). There was also a proliferation of single melanocytes in the epidermis, with lentiginous hyperplasia and single melanocytes present in the spinous and granular cell layers in pagetoid extension. Dermal melanocytes formed asymmetric and expansile nests, and there was a lack of maturation toward the base. Dermal melanocytes showed pleomorphism with some dermal mitoses (Fig 2, *B*). Together, these findings confirmed melanoma. Contiguous with the melanoma was a dermal-based proliferation of delicate wavy spindle cells in a pale pink stroma, representing a dermal neurofibroma. Immunostaining was positive for SOX10 in the melanoma and in the spindle cells of the contiguous neurofibroma (Fig 2, *C*), while MART-1 was positive only in the melanocytic proliferation (Fig 2, *D*). Biopsy confirmed an initial diagnosis of a right retroarticular melanoma at least 2.1 mm Breslow depth, without ulceration, arising in association with a neurofibroma.

Wide local excision was performed with 2 cm margins and subsequent sentinel node biopsy. The postoperative pathology report revealed a superficial spreading melanoma that had arisen within a diffuse dermal neurofibroma present at the deep and peripheral margins. Sentinel lymph node biopsy was positive in 1/6 lymph nodes, which showed melanoma at the right cervical level with extracapsular extension. After excision, the melanoma measured 7.5 mm Breslow depth with the staging of pT4bN1M1. Genetic testing revealed the melanoma was BRAF-negative. Subsequent positron emission tomography-computed tomography imaging showed concerning findings for metastases in the liver and bones. Currently, the patient is followed by oncology and treated with monthly courses of nivolumab.

## Discussion

Here, we report a rare case of melanoma developing in an existing cNF in an individual with NF1. In the case of this patient, her sun exposure and history of melanoma in situ may have increased her risk of melanoma in a sun-exposed region. While NF1 patients have a higher rate of tumor development than the general population, the tumors with the highest penetrance are typically benign. While malignant melanoma is a relatively common tumor in the general population, studies have not demonstrated a clear elevated risk for patients with NF1.^3^ Some studies have linked melanocytic neoplasms and neurofibromatosis in NF1 patients, but other retrospective analyses have not revealed substantial evidence of this association.^2^, ^3^, ^4^ However, in a broad retrospective analysis of individuals with NF1, patients who did develop tumors, including melanoma, developed them at a younger age and had lower disease-specific survival when compared to the general population.^1^

The *NF1* gene is frequently mutated in sporadic human tumor types including melanoma.^6^^,^^7^ Somatic *NF1* mutations have been reported in 12% to 30% of cutaneous melanoma and 45% to 90% of desmoplastic melanoma.^7^ Additionally, most melanoma is associated with upregulation of RAS signaling with other common tumor-activating mutations including neuroblastoma ras viral oncogene homolog and BRAF.^6^^,^^7^ Given that *NF1* loss and overactivation of RAS signaling drives tumorigenesis in these contexts, it would be expected that more patients with NF1 would develop melanoma, along with other Ras-driven malignancies.^8^ In the case of benign cNFs and café-au-lait macules, both highly penetrant lesions in NF1, neither Schwann cells nor melanocytes undergo malignant transformation, despite having biallelic mutations in the *NF1* gene. Evidence from NF1 mouse models suggested that the *NF1-*heterozygous microenvironment is responsible for promoting benign tumor formation while preventing malignant transformation.^9^ It was suggested that this phenomenon may be due to increased immune surveillance in the heterozygous tumor suppressor condition as loss of 1 copy of the *NF1* gene is sufficient to enhance T cell proliferation and function.^8^, ^9^, ^10^ Melanoma is an immune-responsive tumor, thus in an *NF1-*heterozygous context, this malignancy would be less likely to develop.

While melanoma can result from a pre-existing nevus, it is rare to encounter a melanoma arising in the same location as a pre-existing neurofibroma. There has only been 1 reported case in the literature of melanocytic lesions developing at the same site as a neurofibroma within the skin of a non-NF1 patient.^5^ In that case, the non-NF1 patient, who had a history of non-melanoma skin cancer, developed melanoma at the site of a cluster of cNFs. The current case involves a patient with a confirmed diagnosis of NF1 who developed melanoma in the same location as a documented cNF. It is possible that *NF1* loss and subsequent increased ras/mitogen-activated protein kinase signaling in the cNF led to increased proliferation of surrounding melanocytes that ultimately developed the melanoma. Though reportedly an uncommon occurrence, this case emphasizes the need for regular skin examinations and thorough work-up of atypical nevi in NF1 patients, especially when the lesion is growing or changing rapidly.

## Conflicts of interest

None disclosed.
